# Biochemical Properties of a Novel Cysteine Protease of *Plasmodium vivax*, Vivapain-4

**DOI:** 10.1371/journal.pntd.0000849

**Published:** 2010-10-12

**Authors:** Byoung-Kuk Na, Young-An Bae, Young-Gun Zo, Youngchool Choe, Seon-Hee Kim, Prashant V. Desai, Mitchell A. Avery, Charles S. Craik, Tong-Soo Kim, Philip J. Rosenthal, Yoon Kong

**Affiliations:** 1 Department of Molecular Parasitology and Center for Molecular Medicine, Samsung Biomedical Research Institute, Sungkyunkwan University School of Medicine, Suwon, Korea; 2 Department of Parasitology and Institute of Health Sciences, Gyeongsang National University School of Medicine, Jinju, Korea; 3 Department of Pharmaceutical Chemistry, University of California San Francisco, San Francisco, California, United States of America; 4 Department of Medicinal Chemistry, National Center for Natural Products Research, University of Mississippi, University, Mississippi, United States of America; 5 Department of Chemistry and Biochemistry, University of Mississippi, University, Mississippi, Unites States of America; 6 Department of Parasitology, Inha University College of Medicine, Incheon, Korea; 7 Department of Medicine, University of California San Francisco, San Francisco, California, United States of America; McGill University, Canada

## Abstract

**Background:**

Multiple cysteine proteases of malaria parasites are required for maintenance of parasite metabolic homeostasis and egress from the host erythrocyte. In *Plasmodium falciparum* these proteases appear to mediate the processing of hemoglobin and aspartic proteases (plasmepsins) in the acidic food vacuole and the hydrolysis of erythrocyte structural proteins at neutral pH. Two cysteine proteases, vivapain (VX)-2 and VX-3 have been characterized in *P. vivax*, but comprehensive studies of *P. vivax* cysteine proteases remain elusive.

**Findings:**

We characterized a novel cysteine protease of *P. vivax*, VX-4, of which orthologs appears to have evolved differentially in primate plasmodia with strong cladistic affinity toward those of rodent *Plasmodium*. Recombinant VX-4 demonstrated dual substrate specificity depending on the surrounding micro-environmental pH. Its hydrolyzing activity against benzyloxycarbonyl-Leu-Arg-4-methyl-coumaryl-7-amide (Z-Leu-Arg-MCA) and Z-Phe-Arg-MCA was highest at acidic pH (5.5), whereas that against Z-Arg-Arg-MCA was maximal at neutral pH (6.5–7.5). VX-4 preferred positively charged amino acids and Gln at the P1 position, with less strict specificity at P3 and P4. P2 preferences depended on pH (Leu at pH 5.5 and Arg at pH 7.5). Three amino acids that delineate the S2 pocket were substituted in VX-4 compared to VX-2 and VX-3 (Ala90, Gly157 and Glu180). Replacement of Glu180 abolished activity against Z-Arg-Arg-MCA at neutral pH, indicating the importance of this amino acid in the pH-dependent substrate preference. VX-4 was localized in the food vacuoles and cytoplasm of the erythrocytic stage of *P. vivax*. VX-4 showed maximal activity against actin at neutral pH, and that against *P. vivax* plasmepsin 4 and hemoglobin was detected at neutral/acidic and acidic pH, respectively.

**Conclusion:**

VX-4 demonstrates pH-dependent substrate switching, which might offer an efficient mechanism for the specific cleavage of different substrates in different intracellular environments. VX-4 might function as a hemoglobinase in the acidic parasite food vacuole, a maturase of *P. vivax* plasmepsin 4 at neutral or acidic pH, and a cytoskeleton-degrading protease in the neutral erythrocyte cytosol.

## Author Summary

*Plasmodium vivax* affects hundreds of millions each year and results in severe morbidity and mortality. Plasmodial cysteine proteases (CPs) play crucial roles during the progression of malaria since inhibition of these molecules impairs parasite growth. These CPs might be targeted for new antimalarial drugs. We characterized a novel *P. vivax* CP, vivapain-4 (VX-4), which appeared to evolve differentially among primate *Plasmodium* species. VX-4 showed highly unique substrate preference depending on surrounding micro-environmental pH. It effectively hydrolyzed benzyloxycarbonyl-Leu-Arg-4-methyl-coumaryl-7-amide (Z-Leu-Arg-MCA) and Z-Phe-Arg-MCA at acidic pH and Z-Arg-Arg-MCA at neutral pH. Three amino acids (Ala90, Gly157 and Glu180) that delineate the S2 pocket were found to be substituted in VX-4. Alteration of Glu180 abolished hydrolytic activity against Z-Arg-Arg-MCA at neutral pH, indicating Glu180 is intimately involved in the pH-dependent substrate preference. VX-4 hydrolyzed actin at neutral pH and hemoglobin at acidic pH, and participated in plasmepsin 4 activation at neutral/acidic pH. VX-4 was localized in the food vacuoles and cytoplasm of the erythrocytic stage of *P. vivax*. The differential substrate preferences depending on pH suggested a highly efficient mechanism to enlarge biological implications of VX-4, including hemoglobin degradation, maturation of plasmepsin, and remodeling of the parasite architecture during growth and development of *P. vivax*.

## Introduction

*Plasmodium vivax*, one of the most predominant human malaria species worldwide, causes hundreds of millions of illnesses each year, and can result in severe morbidity and mortality, especially in children [1], [2]. Emergence and spread of multidrug resistant vivax malaria is an increasing problem, which is associated with fatal disease [3]–[5].

Cysteine proteases of malaria parasites are intimately involved in a variety of physiological processes essential for the parasite's survival. The potential roles of the cysteine proteases of *P. falciparum* such as falcipain-2 (FP-2), FP-2B (synonym of FP-2′) and FP-3 in hemoglobin degradation in the acidic parasite food vacuole [6], [7], processing of food vacuole plasmepsins to active proteases [8], and erythrocyte rupture *via* cleavage of cytoskeletal proteins followed by merozoite release [9] have been well characterized. Genes encoding three closely related FPs cluster on chromosome 11 within a 12-kb stretch, called the cysteine protease island [10]. FP-2 and FP-2B share similar primary structure and enzymatic properties [11]. Knockout of the FP-2 gene leads to a transient block in hemoglobin hydrolysis, but parasites compensate for the loss through production of FP-2B and/or FP-3, and they multiply at the same rate as wild type parasites. In contrast, FP-3 appears to have an essential role, as knockout of the FP-3 gene is lethal [12]. Another papain-like cysteine protease FP-1, of which gene locates on chromosome 14, appears to be active in early invasive merozoites and in oocyst production in mosquitoes [13], [14]. *Plasmodium* cysteine proteases appear to display non-overlapping roles with differentiated biochemical properties and expression patterns, as well as redundancy and complementation [15].

Two cysteine proteases, vivapain (VX)-2 and VX-3, which are encoded on chromosome 9, have been identified in *P. vivax* [16], [17]. VX-2 and VX-3 share a number of biochemical properties with FP-2 and FP-3, including acidic pH optima, requirement for reducing conditions for maximal enzyme activity, and preference toward peptide substrates with positively charged residues at the P1 position and Leu at P2 [17]. Structural modeling of VX-2 and VX-3 has also revealed a topology similar to those of FP-2 and FP-3; however, some substantial differences are detected in the predicted sizes of the binding pockets and residues involved in substrate binding [18]. A gene (XM_001612308) encoding a protein with significant similarity to FP-1 has recently become available in the nucleotide sequence of *P. vivax* (PVX_195290, PVX_239290, and PVX_240290); its biochemical properties and biological activity remain unclear.

Interests in specific inhibitors impeding plasmodial cysteine proteases have focused on their chemotherapeutic applicability; effective inhibitors impair normal parasite growth *in vitro* [19]. In addition, rupture of the erythrocyte membrane by mature parasites is inhibited by broad-spectrum inhibitors of serine and cysteine proteases [20], [21]. Identification and further characterization of *P. vivax* cysteine proteases will be helpful to investigate their biological roles and to characterize targets for antimalarial drugs. However, comprehensive studies of *P. vivax* cysteine proteases have been hindered by an inability to culture *P. vivax*.

In the present study, we describe the biochemical properties of a novel cysteine protease of *P. vivax*, designated vivapain-4 (VX-4), which displays unusual pH-dependent substrate specificity. Molecular modeling and subsequent mutation analysis demonstrated that Glu180 is involved in the pH-dependent substrate specificity of VX-4. The protease effectively hydrolyzed hemoglobin at acidic pH, actin at neutral pH, and plasmepsin 4 at neutral and acidic pHs, supporting its role in the maintenance of metabolic homeostasis and architectural remodeling of the parasite during growth and development.

## Materials and Methods

### Ethics Statement

All animals used in this study were housed in accordance with guidelines from the Association for the Assessment and Accreditation of Laboratory Animal Care (AAALAC). All protocols were approved by the Institutional Review Board and conducted in the Laboratory Animal Research Center of Sungkyunkwan University.

### *In silico* Identification of Cysteine Protease Gene

Genes putatively coding for cysteine proteases were identified from primate and rodent *Plasmodium* sequences deposited in PlasmoDB (http://plasmodb.org) and GenBank (http://www.ncbi.nlm.nih.gov/) through BLAST searches. The amino acid (AA) sequences of cysteine proteases of *P. falciparum* (FP-1, FP-2, FP-2B, and FP-3), *P. vivax* (VX-2 and VX-3), *P. yoelii* (yoelipain [YP]-1 and YP-2), and *P. berghei* (bergheipain [BP]-1 and BP-2) were used in multiple queries, with a threshold at 0.001 (*E*-value cut-off). After excluding redundancies, the AA sequences were aligned with ClustalX and optimized with GeneDoc. The alignment was used as an input in the construction of neighbor joining and maximum likelihood trees using PHYLIP (ver. 3.6b) and TREE_PUZZLE (ver. 5.2). The standard error in each of the connecting nodes was estimated by bootstrapping of 1000 replicates. Two novel cysteine proteases isolated from *P. vivax* were annotated as *P. vivax* cysteine protease 1 (VX-1; XP_001615807) and 4 (VX-4; XP_001615272), according to their clustering patterns in the trees.

### Expression and Refolding of a Recombinant VX-4 (rVX-4)

The open reading frame (ORF) of *VX-4* was amplified with forward (5′- ATGGAATATCACATGGAGTACTCGAAC-3′) and reverse (5′-CTAGTCAAGCAGGGGGACGTACGCCTC-3′) primers using Ex *Taq* DNA polymerase (Takara) and *P. vivax* genomic DNA (100 ng) isolated from a Korean patient (a generous gift from Dr. JS Yeom). The product was gel-purified, ligated into the pCR2.1 vector (Invitrogen) and transformed into competent *E. coli* Top10 cells (Invitrogen). The nucleotide sequence was determined with an ABI PRISM 377 DNA sequencer (Applied Biosystems). The DNA fragment harboring the mature region and a portion of the prodomain from AA position 182 were amplified using 2 primers; 5′-GAGCTCGAGATGCAGCAGAGGTACCT-3′ (contains a 5′ *Sac* I site) and 5′-CTGCAGCTAATCCACGAGCGCAACGA-3′ (contains a 5′ *Pst* I site). The PCR product was ligated and transformed as described above, and ligated into the pQE-30 expression vector (Qiagen). The plasmid was transformed into competent *E. coli* M15 (pREP4) cells (Qiagen), grown overnight in LB medium and induced with 1 mM isopropyl-1-thio-β-D-galactopyranoside for 3 h at 37°C. The bacterial cells were suspended in lysis buffer and then centrifuged. rVX-4 was purified from the supernatant by nickel-nitrilotriacetic acid (Ni-NTA, Qiagen) chromatography, following the manufacturer's instruction. Optimal refolding conditions for rVX-4 were determined with 100 different buffer combinations in a microplate format [22]. For large-scale refolding, purified rVX-4 (100 mg) was diluted 100-fold in optimized refolding buffer (250 mM L-arginine, 1 mM ethylenediaminetetraacetic acid [EDTA], 5 mM reduced glutathione [GSH], 1 mM oxidized glutathione [GSSG], and 100 mM Tris-HCl, pH 8.0), and incubated overnight at 4°C. To allow processing to the active enzyme, the pH was adjusted to 5.5 in the presence of 10 mM dithiothreitol (DTT), the sample was incubated at 37°C for 2 h, and the pH was then readjusted to 6.5. The protein was concentrated with a Centriprep concentrator (cut-off: 10 kDa, Millipore).

### N-terminal AA Sequencing

The fully processed rVX-4 was separated by 12% SDS-PAGE. The protein was transferred to a polyvinylidene difluoride (PVDF) membrane (Millipore) and stained with Coomassie blue. The band was excised and subjected to protein sequencing on an ABI model 477A protein sequencer and an ABI model 120A PTH analyzer (Applied Biosystems) at the Korea Basic Science Institute (Daejeon, Korea).

### Specific Antibodies

Six-wk-old, specific pathogen free (SPF) BALB/*c* female mice were subcutaneously immunized 3 times with the purified rVX-4 (30 µg per each mouse per each time) in Freund's adjuvants (Sigma-Aldrich) at 2-wk intervals. One week after the final inoculation, 10 µg protein were injected *via* tail vein. One week later, the blood was collected by heart puncture, after which the antiserum was prepared. BALB/*c* mouse (6-wk-old) serum obtained from SPF strain was used as a normal control.

### Cysteine Protease Activity Assay and Kinetics

Cysteine protease activity was ascertained by the hydrolysis of benzyloxycarbonyl-L-leucyl-L-arginine 4-methyl-coumaryl-7-amide (Z-LR-MCA) (Peptide International). Enzyme (30 µl; 200 nM) was added to 100 mM sodium acetate (220 µl, pH 5.5) containing 5 µM Z-LR-MCA and 10 mM DTT. The release of fluorescence was assessed at excitation and emission wavelengths of 355 nm and 460 nm with a SpectraMAX Gemini fluorometer (Molecular Devices). For activity gel electrophoresis, refolded rVX-4 was mixed with SDS-PAGE sample buffer lacking 2-mercaptoethanol and subjected to 12% SDS-PAGE co-polymerized with 0.1% gelatin. The gel was washed with 2% Triton X-100 (30 min), incubated overnight with 100 mM sodium acetate (pH 5.5) containing 10 mM DTT at 37°C and stained with Coomassie Blue. For kinetic analysis, the rVX-4 (25 nM) was incubated with varying concentrations of peptide substrates at pH 5.5, 6.5 and 7.5 in appropriate buffers, each supplemented with 10 mM DTT. The release of MCA was monitored over 10 min at room temperature as described above. Activities were compared as fluorescence over time. The kinetic constants *K*_m_ and *V*_max_ were determined using PRISM (GraphPad Software). The optimal pH was assessed in 100 mM sodium acetate (pH 4.5–5.5), 100 mM sodium phosphate (pH 6.0–6.5) and 100 mM Tris-HCl (pH 7.0–8.5). The enzyme (50 nM) was added to each buffer supplemented with 10 mM DTT and 5 µM Z-L-phenyl-L-arginine 4-methyl-coumaryl-7-amide (Z-FR-MCA), Z-leucyl-L-arginine-MCA (Z-LR-MCA), or Z-L-arginyl-L-arginine 4-methyl-coumaryl-7-amide (Z-RR-MCA). The appropriate buffers were separately employed as controls at each pH. Enzyme activity was measured as described above. The effects of reducing agents were examined under various concentrations of GSH, and pH stability was examined at pH 5.0 and 8.0 by incubating rVX-4 at 37°C in the appropriate buffer. Active site titration was done using a specific inhibitor, *trans*-epoxysuccinyl-L-leuciloamido-(4-guanidino) butane (E-64).

### Positional Scanning of Tetrapeptide Substrate Libraries

Two synthetic combinatorial libraries were used to determine the substrate specificities of the S1–S4 subsite of rVX-4. To determine P1 specificity, a P1 diverse library consisting of 20 sublibraries was employed. In each sublibrary, the P1 position contained one native AA, and the P2, P3, and P4 positions were randomized with equimolar mixtures of AAs for 6859 tetrapeptide substrates sequenced per sublibrary (in each case, cysteine was omitted and methionine was replaced by norleucine). A total of 20 aliquots (5×10^−9^ M) of each sublibrary were dispensed into wells of a 96-well microfluor-1 U-bottom plate (Dynex) at a final concentration of 7.3 nM. To determine P2, P3, and P4 specificity, a complete diverse library was used in which the P2, P3, or P4 position was spatially addressed with 20 AAs (norleucine was substituted for cysteine) and the remaining 3 positions were randomized. Aliquots (2.5×10^−8^ M) from each sublibrary were added to 60 wells of a 96-well microfluor-1 U-bottom plate. Each well contained 8000 compounds (final concentration of 30 nM). Hydrolytic reactions were initiated by the addition of rVX-4 (10 nM) and monitored fluorometrically as described above. Assays were performed at 37°C in 100 mM sodium acetate (pH 5.5), 100 mM sodium phosphate (pH 6.5), or 100 mM Tris-HCl (pH 7.5), in each case with 100 mM NaCl, 10 mM DTT, 1 mM EDTA, 0.01% Brij-35 and 1% dimethylsulfoxide (DMSO).

### Hydrolysis of Macromolecular Substrates

To observe possible roles of VX-4 in the processing of plasmepsin (PM), we cloned *P. vivax* plasmepsin (PvPM) 4 (XM_001616821) employing *P. vivax* genomic DNA obtained from the Korean patient as previously described [23]. Recombinant PvPM4 expressed in *E. coli* cells was purified by Ni-NTA chromatography (Qiagen) and refolded as described above. rVX-4 (50 nM) was incubated with PvPM4 (20 µg) in 100 mM sodium acetate (pH 5.0–5.5), 100 mM sodium phosphate (pH 6.0–6.5), or 100 mM Tris-HCl (pH 7.0–7.5) supplemented with 10 mM DTT for 3 h. The experiments were also performed in the presence of E-64 (1 µM) and/or pepstatin A (10 µM). Hemoglobinase activity of rVX-4 (30 nM), as well as those of rVX-2 and rVX-3 expressed as previously described [17], was assessed using human hemoglobin (Sigma-Aldrich) in different pHs (5.0–7.5) in the presence of 1 mM GSH at 37°C. Erythrocyte ghosts purified from fresh human blood by hypotonic lysis were incubated with rVX-4 (200 nM) at pH 7.0 or 7.5 at 37°C for 3 h, after which reaction products were analyzed by SDS-PAGE. For immunoblotting, the electrophoretically resolved proteins were transferred to PVDF membranes (Millipore) followed by blocking with 0.05% Tween 20 in phosphate buffered saline (PBST) containing 2% bovine serum albumin. The membrane was incubated with appropriate antibodies including anti-human spectrin (Sigma-Aldrich, 1∶500 dilutions), anti-human band 3 (Sigma-Aldrich, 1∶3000 dilutions), or anti-human actin (Sigma-Aldrich, 1∶1000 dilutions). Blots were subsequently incubated with horseradish peroxidase-conjugated host specific antibodies. The immunoreactive bands were visualized using 4-chloro-1-naphthol (4C1N; Sigma-Aldrich) supplemented with 3% hydrogen peroxide.

### Comparative Protein Structure Modeling

Computational analyses were accomplished in a Silicon Graphics Octane 2 workstation, equipped with two parallel R12000 processors (SGI). Homology modeling was orchestrated within the SYBYL 6.9 COMPOSER module (Tripos Associates, MO). Energy minimization and molecular dynamic studies were performed with the DISCOVER module of InsightII 2000 (Accelrys). The geometrical and local environmental consistency of the model was assessed within the PROSTAT and InsightII 2000 Profiles-3D modules, together with the SYBYL 6.9 Matchmaker module. Structural models of FP-2, FP-3, VX-2, VX-3 and VX-4 mature domains were prepared on the basis of their sequence homology with several cysteine proteases using an analogous approach [18]. More than 35% sequence identity was observed between the protein homologs and the target AA sequence. The homologs used in this analysis included human cathepsins K (1ATK), V (1FH0) and S (1MS6); cruzain (1AIM), a cysteine protease from *Ginger rhizome* (1CQD) and actinidin (1AEC). Terms in parentheses refer to the Protein DataBank accession numbers for the corresponding crystal structures.

### Mutation Analyses

Site-directed mutagenesis was performed using a QuickChange II Site-Directed Mutagenesis Kit (Stratagene). A pair of complementary primers with 39 bases was designed and a mutation to replace Ala90 to Ile (A90I), Gly154 to Ser (G154S) or Glu180 to Ala (E180A) was placed in the middle of the primers. Parental DNA inserted in pQE-30 was amplified using *Pfu* Ultra HF DNA polymerase with these primers for 16 cycles in a DNA thermal cycler (Perkin-Elmer). After digestion of the parental DNA with *Dpn* I, the amplified DNA with nucleotide substitution was incorporated and transformed into *E. coli* XL1-Blue (Stratagene). The mutations were verified by DNA sequencing. Double and triple point mutagenesis of A90I, G154S, and E180A were also done as described above. Each mutant plasmid was transformed into competent *E. coli* M15 (pREP4) cells (Qiagen). Each recombinant protein was individually expressed, purified and refolded as described above.

### Immunocytochemical Staining

Thin blood smears (2 µl) were prepared from EDTA-containing venipuncture blood immediately after sampling from patients infected with *P. vivax* (gift from Dr. JS Yeom). A part of the slides were stained with 3% Giemsa, rinsed and air dried. The unstained thin films were treated with 3% H_2_O_2_ for 5 min and incubated with 1% bovine serum albumin. The films were incubated with mouse anti-rVX-4 antibody (1∶500 dilutions in PBS). The reactions were visualized with an avidin-biotin complex (DAKO) and examined under a light microscope (Axiophot, Carl Zeiss).

## Results and Discussion

### The *P. vivax* Genome Encodes Four Closely Related Vivapains

**Figure 1:**
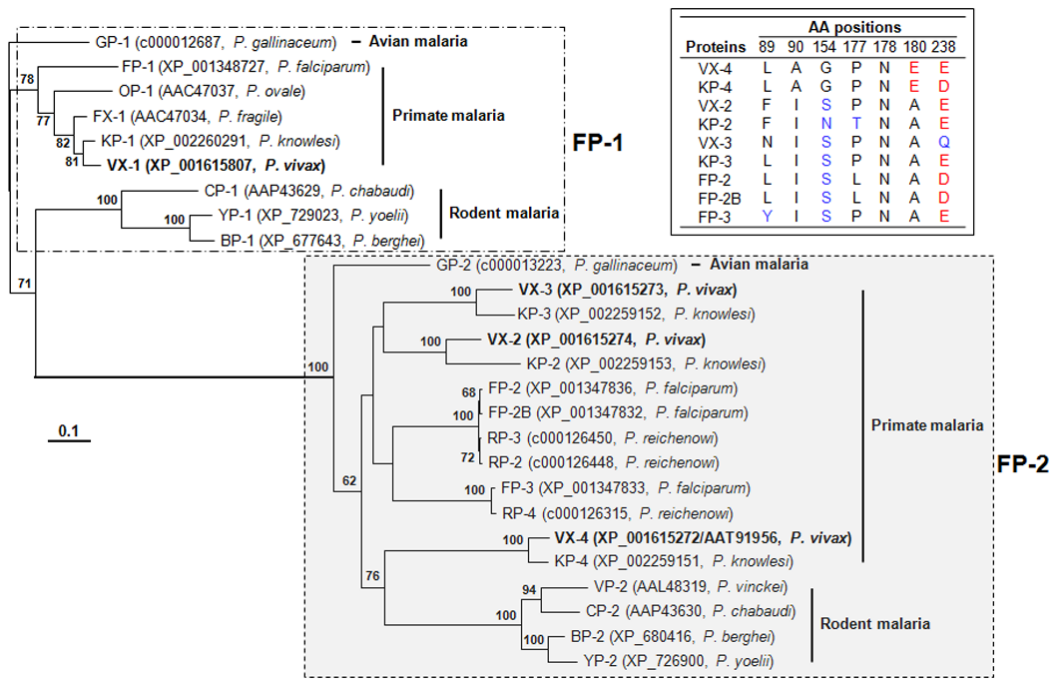
Phylogenetic analysis of malaria cysteine proteases including VX-4. The phylogeny was based on the AA sequence alignment of mature regions. Divergence rates were calculated with the Jones-Taylor-Thornton (JTT) substitution model, and the tree was constructed using the neighbor joining algorithm. The tree was rooted with GP-1 of *Plasmodium gallinaceum*, which was taken as an out-group. The number at each of the branching nodes indicates the likelihood (percentage) of its appearance in the bootstrapping analysis with 1000 replicates. The enzymes from *P. vivax* are in bold. The box indicates AAs found in the S2 pocket of primate plasmodial proteases, with position numbers based on mature VX-4. Red, blue, and black AAs are acidic, uncharged polar and hydrophobic, respectively. Note: The vivax protein with accession no. XP_001615272 was annotated as VX-2 during primary analysis of the whole genome sequence of the *P. vivax* Sal I strain. The name is changed to VX-4 according to our current result (AAT91956).

By data-mining of the *P. vivax* genome (TIGR, Release 2.0), we identified two genes putatively coding for novel cysteine proteases, in addition to the previously identified genes encoding VX-2 (PlasmoDB code PVX_091415) and VX-3 (PVX_091410). We designated these genes as *VX-1* (PVX_195290) and *VX-4* (PVX_091405). The other primate *Plasmodium* genomes examined, such as *P. falciparum*, *P. reichenowi* and *P. knowlesi*, also harbored four closely related cysteine protease genes. Conversely, avian and rodent malaria parasites including *P. gallinaceum*, *P. yoelii*, and *P. berghei* possessed only two paralogous genes (Figure 1). The deduced AA sequence of VX-4 (TC5625, 484 AAs) revealed considerable degrees of identity to that of VX-2 (TC5622, 59%) and VX-3 (TC5618, 48%), while that of VX-1 (TC5613, 583 AAs) was highly related to the FP-1-like proteases of *P. falciparum*, *P. knowlesi*, *P. ovale* and *P. fragile* (37–77% identity). The greater length of VX-1 might be attributable to an N-terminal extension [24]. Physiological implications and specific domain(s)/signature(s) of VX-1 remain largely elusive. The primary structure of VX-4 tightly conserved the AA residues lining the catalytic site (Gln, Cys, His, Asn and Trp) that are essential for the stabilization of a thiolate-imidazolium ion pair and/or the transition state of the catalytic site (AA positions highlighted in blue in Supplementary Figure S1). The regulatory motifs of the plasmodial cysteine proteases such as a bipartite trafficking domain, inhibitor domain with ERFNIN signature and hemoglobin-binding FP2 arm were also clearly identified in each of the corresponding regions (Supplementary Figure S1) [25], [26]. The eight Cys residues, which are involved in the maintenance of structural geometry, were well conserved in these proteins, whereas the last Cys was replaced by Asn in VX-4 and KP-4 (arrowheads in Supplementary Figure S1). Given the fact that a disulfide bridge between the seventh and eighth Cys residues is intimately engaged in the stabilization of the S2 and S1′ sites of FP-2 [27], the more flexible binding pocket of VX-4 might allow broader accessibility of proteolytic substrates. In addition, several AA substitutions found in critical domains of VX-4 suggest a distinctive physiological role for this protease (Supplementary Figure S1). These collective data demonstrate that VX-4 is a distinct cysteine protease that shares significant identity with, but clearly differs from previously characterized *P. vivax* cysteine proteases.

### *Plasmodium* Cysteine Proteases Exhibit Differential Evolutionary Episodes Along with Their Donor Organisms

A neighbor-joining tree of VX-1 and VX-4 homologs, which were retrieved from PlasmoDB and GenBank, was constructed employing the AA sequences of mature domains (Figure 1). The *Plasmodium* proteases were largely separated into two distinct clusters consistent with their predicted biological roles: FP-1 clade, of which members are implicated in host cell invasion [13] and oocyst production [14], and FP-2 clade, the majority of which play central roles in hemoglobin degradation [6], [11], [28]. An overall topology similar to that of the neighbor-joining tree was observed in a quartet maximum likelihood tree (TREE_PUZZLE program; data not shown) and the major branching nodes were supported by significant bootstrapping or quartet values. The falcipain homolog genes appeared to have duplicated from a common ancestor before diverging into each of the avian and mammalian parasite lineages. The FP-1 family proteins seemed to have diverged along with their specific donor organisms without any provocative genetic event. Meanwhile, members of FP-2 clade might have more complicated evolutionary pathways, including either multiplication(s) in primate malaria or deletion(s) in rodent malaria. The genes orthologous to *VX-2* and *VX-3* may have been deleted in the rodent parasites, considering the polytomic relationships among the *P. vivax* and *P. knowlesi* paralogs and the tight clustering of VX-4/KP-4 with rodent malarial proteins. This suggestion is further supported by the fact that *P. falciparum* and *P. reichenowi*, which comprise a basal clade in mammalian *Plasmodium* lineages [29], [30], contain three paralogous genes. The three paralogous genes occupying distinct but highly linked genomic loci (cysteine protease island) may have undergone a kind of convergent evolution events in these basal malaria genomes.

Adding to increased genic dosage, the degree of sequence divergence was prominent among the primate FP-2 clade members (0.812±0.078), compared to related rodent proteins (0.271±0.034). The members of primate (0.266±0.035) and rodent (0.377±0.056) FP-1 clade displayed values similar to that of the rodent FP-2-like proteins (Supplementary Table S1). Alteration in gene copy number provides a simple way to change expression levels or to enlarge protein pools with non-overlapping functions. Biochemical studies have demonstrated that the primate malaria proteins belonging to the FP-2 clade exhibit similar enzymatic properties; however, those of *P. vinckei* (VP-2) and *P. berghei* (BP-2) demonstrated quite dissimilar features, particularly in terms of their substrate preference and inhibitor specificity [31],[32]. Therefore, the large divergence among the primate FP-2 proteins and tight clustering of VX-4 and KP-4 with rodent *Plasmodium* proteins (bootstrapping value 76) further suggest biological roles of VX-4 that are distinct from those previously described for VX-2 and VX-3 [16], [17].

### Recombinant VX-4 Shows Different Substrate Preferences Depending on pH

**Figure 2:**
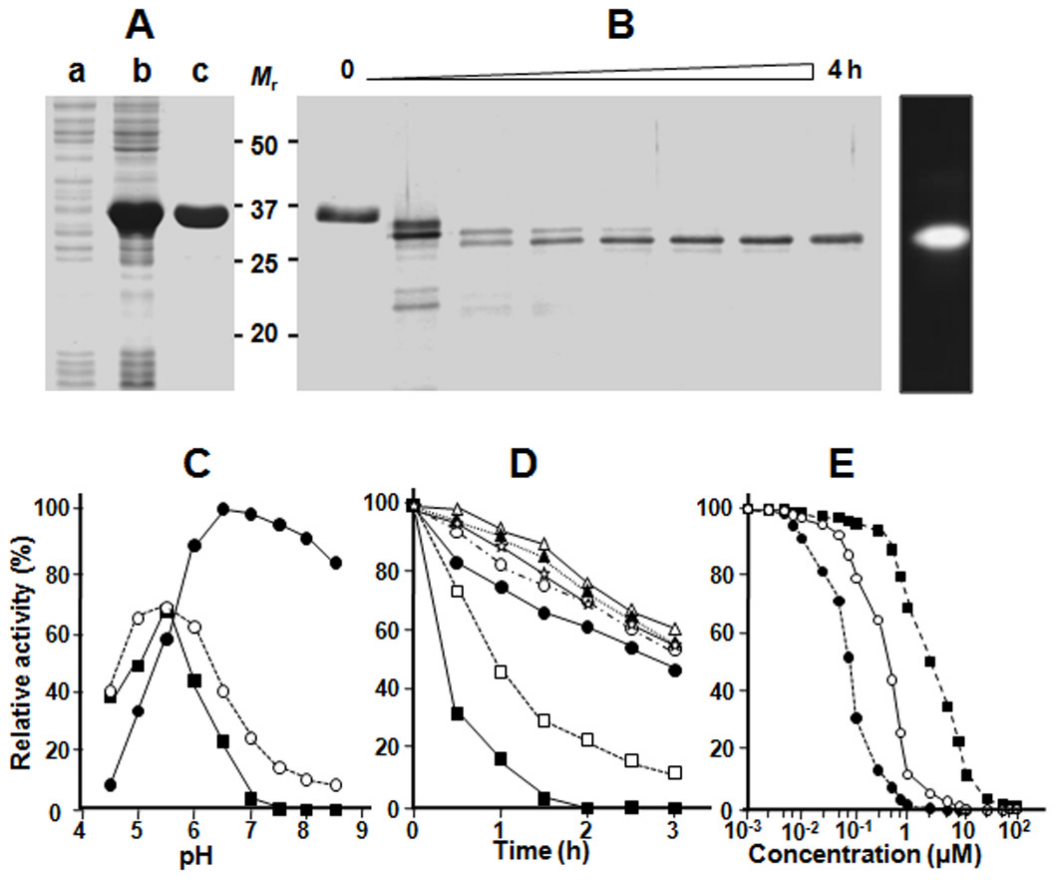
Biochemical properties of recombinant VX-4. (**A**) Expression and purification of rVX-4. Proteins were analyzed by 12% SDS-PAGE and stained with Coomassie blue. Lanes a, uninduced *E. coli* lysate; b, IPTG-induced *E. coli* lysate; c, Ni-NTA purified rVX-4. *M*_r_, molecular masses in kDa. (**B**) Processing of the refolded rVX-4. The purified rVX-4 was refolded and activated, and aliquots collected every 30 min were analyzed by 12% SDS-PAGE with Coomassie staining (left). The proteolytic activity of fully processed rVX-4 was analyzed by a gelatin gel-zymogram (right). (**C**) Determination of pH optimum. The VX-4 enzyme activity was assayed in 100 mM sodium acetate (pH 4.5–5.5), sodium phosphate (pH 6.0–6.5) or Tris/HCl (pH 7.0–8.5), each supplemented with 10 mM DTT. Activity was measured at 37°C each against Z-FR-MCA (▪), Z-LR-MCA (○), and Z-RR-MCA (•). Maximal activity was presented as 100%. (**D**) Determination of enzyme stability. rVX-4 was incubated at different pHs in the respective buffers as in panel C. Residual activity was assayed with Z-LR-MCA in 100 mM sodium acetate (pH 5.5) supplemented with 1 mM DTT after indicated incubations at pH 4.5 (•), 5.0 (○), 5.5 (▴), 6.0 (▵), 7.0 (⋆), 8.0 (□) and 8.5 (▪). (**E**) Inhibition profile for E-64 was determined by incubating rVX-4 (1 µM) with different concentrations of E-64 in 100 mM sodium acetate (pH 5.5; •), 100 mM sodium phosphate (pH 6.5; ○) or 100 mM Tris-HCl (pH 7.5; ▪) at room temperature for 30 min. Residual activities (%) were determined using Z-LR-MCA as a substrate.

The full-length VX-4 gene amplified from a Korean *P. vivax* patient's blood displayed nucleotide sequence identical to that of the reference Sal I strain (nucleotide sequence data is available in the GenBank under the accession no. AY584068). A rVX-4 protein comprising a portion of the prodomain and entire mature domain was expressed in *E. coli* (Figure 2A). Purified rVX-4 was refolded followed by maturation under reducing and mild acidic (pH 5.5) conditions. The fully processed 28 kDa protein (left panel, Figure 2B) exhibited protease activity by gelatin-gel electrophoresis (right panel, Figure 2B), which was completely inhibited by the cysteine protease inhibitor E-64 (data not shown). The N-terminal sequence of fully processed rVX-4 was NSPYV (Supplementary Figure S1).

rVX-4 hydrolyzed synthetic dipeptidyl substrates with hydrophobic AA residues at their P2 site such as Z-LR-MCA and Z-FR-MCA under acidic conditions. Activity was highest at pH 5.5. The pH-optimum was substantially different with a substrate containing a basic AA at P2 (Z-RR-MCA) (Figure 2C) with maximal activity at pH 6.5, and activity seen above pH 8. These results suggest either that electrostatic conditions near the S2 site are highly dependent on the surrounding pH or that the geometry of the catalytic site can be changed in a pH-dependent manner. rVX-4 was relatively stable after incubation at acidic and neutral pH, while it was highly unstable under alkaline conditions (pH 8.0 and 8.5) against Z-LR-MCA (Figure 2D), which was similar to results observed for FP-2 and FP-3 [28], [32]. These data suggest that decreased hydrolyzing activity of rVX-4 against Z-LR-MCA and Z-FR-MCA at alkaline conditions might be due to an irreversible change of the protein conformation. The requirement for increased concentrations of E-64 to inhibit rVX-4 at higher pH also supports altered structural geometry as the explanation for altered substrate preference (Figure 2E).

Steady-state kinetic analyses confirmed varied substrate utilization depending on pH (Table 1). rVX-4 showed a similar catalytic efficiency against three peptide substrates at pH 5.5. However, at pH 7.5 *k_cat_/K_m_* against Z-RR-MCA increased 2.6-fold whereas that against Z-LR-MCA decreased 5.5-fold and Z-FR-MCA was not hydrolyzed. The rVX-2 and rVX-3 exhibited much higher *k_cat_/K_m_* values than that of rVX-4 toward Z-LR-MCA at the pH conditions selected, although the optimal pH for rVX-2 was 6.5, rather than 5.5. Interestingly, rVX-2 and rVX-3 could not hydrolyze Z-FR-MCA or Z-RR-MCA. Phe has a large aromatic R group, and it might not fit into the S2 pocket of rVX-2 and rVX-3, which are stabilized by the disulfide bond between the seventh and eighth Cys residues (Supplementary Figure S1).

**Table 1:** Comparison of substrate hydrolysis kinetics for vivapains.

		*k*_cat_/*K*_m_ (s^−1^M^−1^)		
		VX-2^a^	VX-3^a^	VX-4
pH 5.5	Z-FR-MCA	NH^b^	NH	1.55×10^4^
	Z-LR-MCA	7.05×10^5^	8.62×10^4^	1.65×10^4^
	Z-RR-MCA	NH	NH	1.34×10^4^
pH 6.5	Z-FR-MCA	NH	NH	5.52×10^3^
	Z-LR-MCA	7.34×10^5^	6.36×10^4^	8.84×10^3^
	Z-RR-MCA	NH	NH	3.49×10^4^
pH 7.5	Z-FR-MCA	NH	NH	NH
	Z-LR-MCA	4.15×10^5^	6.26×10^3^	3.05×10^3^
	Z-RR-MCA	NH	NH	3.45×10^4^

Activity values for each enzyme represent mean from three independent experiments.

a Values for VX-2 and VX-3 were adopted from previous report (Na et al., 2004).

b NH, no hydrolysis.

**Figure 3:**
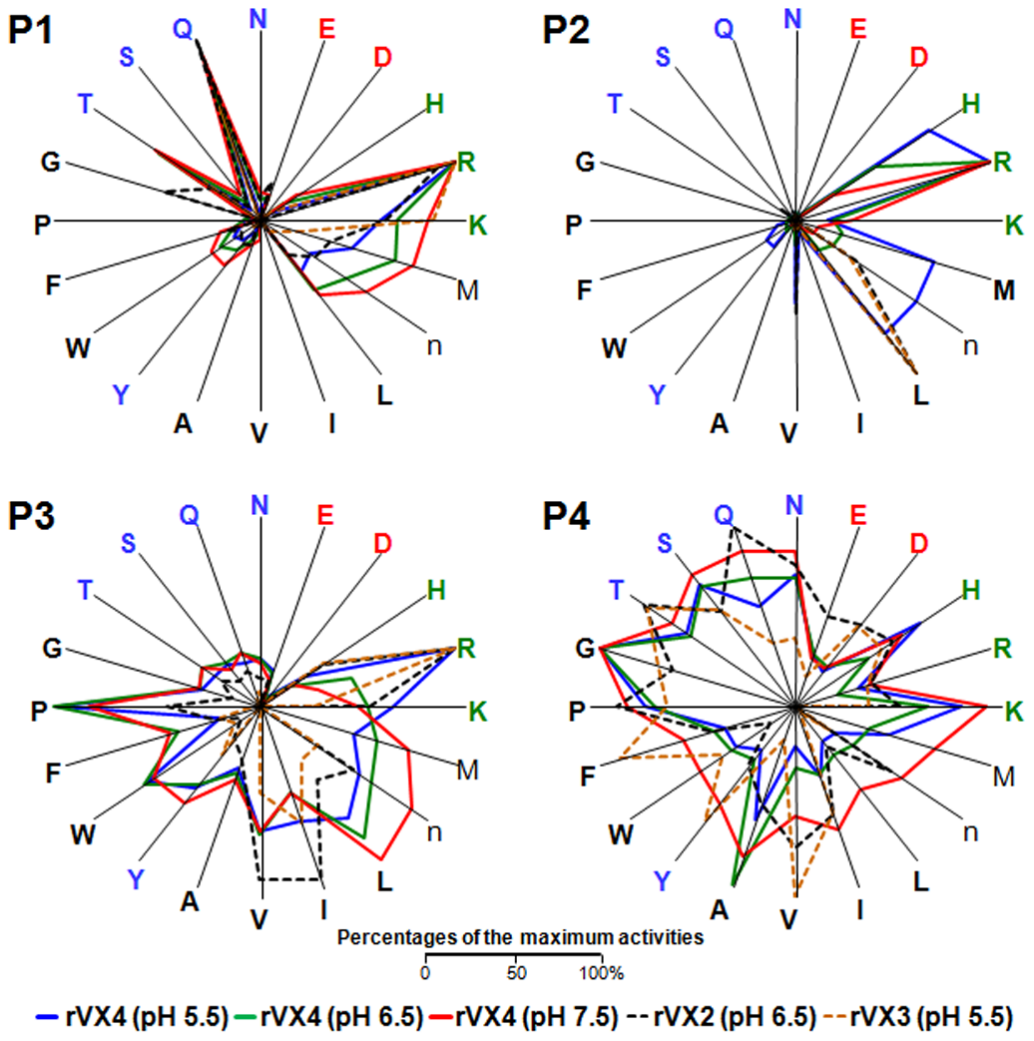
Substrate specificity profiles of vivapains against a tetrapeptide library. A P1 diverse library, and P2, P3, and P4 sublibraries of the P1-lysine fixed library were used to determine specificities. Activities at the four positions (P1, P2, P3 and P4) were compared and are displayed as percentages of the maximum for each position by radial plot. AAs are arranged counter-clockwise from angle zero in decreasing order based on activity of VX-4 at pH 7.5. AAs are represented by the single-letter code (n is norleucine). The representative result of three independent experiments is shown. Black, hydrophobic; Red, acidic; Blue, uncharged polar; Green, basic.

Substrate preferences were further evaluated using a combinatorial tetrapeptide library (Figure 3). AA utilization patterns of vivapains at the P1, P3 and P4 positions were comparable to those of FP-2 and FP-3 [15]. pH changes did not lead to significant alterations in the substrate preference of VX-4 at these sites, except that for tetrapeptides with Arg at P3; VX-4 was active at pH 5.5, with substantial decreases as pH increased. The most striking specificity was observed with substrates with different AAs at P2. The vivapains preferred hydrophobic AAs such as Leu and Val under acidic (VX-3 and VX-4, pH 5.5) and neutral (VX-2, pH 6.5) conditions. VX-4 also exhibited significant activity against P2 Met and His under acidic conditions. However, relative activity against these hydrophobic AA residues was significantly decreased, while that against Arg increased at pH 6.5 and 7.5, consistent with results obtained using synthetic peptide substrates (Table 1).

### Glu180 Contributes to the pH-dependent Substrate Preferences of VX-4

**Figure 4:**
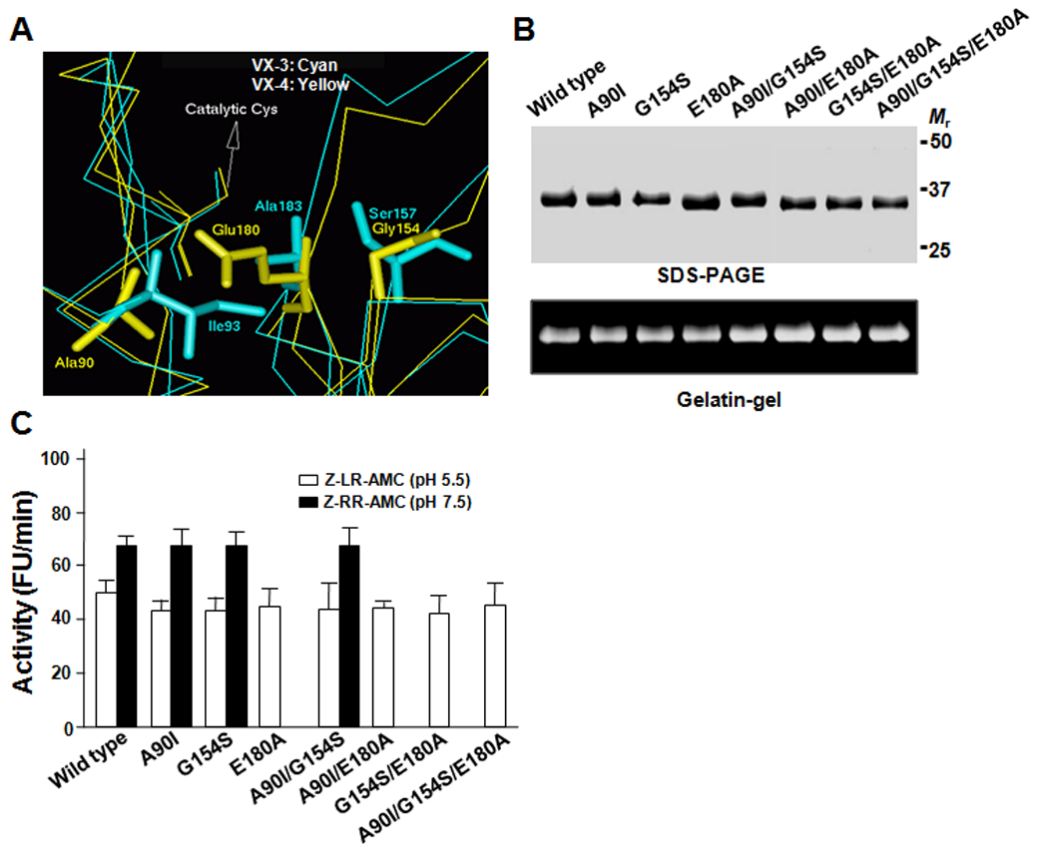
Mutation analyses of VX-4. (**A**) Superimposition of amino acid residues lining the binding pockets in VX-3 (cyan) and VX-4 (yellow). The residues are shown as sticks and the numbers of residues are indicated in reference to the corresponding enzymes. (**B**) Mutagenesis of VX-4. Each mutatant was expressed and analyzed by 12% SDS-PAGE (upper) and by a gelatin substrate gel (lower). (**C**) The activities of wild-type and mutant VX-4s were assayed in 100 mM sodium acetate (pH 5.5) against Z-LR-MCA and 100 mM Tris-HCl (pH 7.5) against Z-RR-MCA at 37°C. In each case, 10 mM DTT was supplemented in reaction buffer. Maximal activity was presented as 100%. Mean ± S.D. (*n* = 3).

Homology modeling of VX-4 demonstrated an overall topology similar to those of FP-2, FP-3, VX-2 and VX-3 with the average pairwise RMSD of 0.98 for the Cα atoms (data not shown). However, a number of substitutions are recognized between VX-4 and the other VXs, including three prominent AA residues delineating the S2 pocket (Ala90, Gly154 and Glu180; numbering from the mature domain of VX-4) (Figure 4A; see also box in Figure 1 and Supplementary Figure S1). The substrate preferences of VX-4 were found to depend on AA residues occupying P2 site and thus, the diagnostic AA substitution might be relevant to the differential biochemistry of VX-4 compared to those of VX-2 and VX-3. Seven mutant forms of VX-4, in which these three AA residues were substituted by single, double, or triple site-directed mutagenesis (A90I, G154S, E180A, A90I/G154S, A90I/E180A, G154S/E180A, and A90I/G154S/E180A), were expressed in *E. coli*, and their proteolytic activities were examined. All of the refolded recombinant proteins showed hydrolytic activity against gelatin (lower panel, Figure 4B). In assays against peptide substrates, all of the mutants harboring A90I and G154S by single or multiple substitutions maintained the pH-dependent substrate specificity of wild-type VX-4. Conversely, those containing E180A lost activity against Z-RR-MCA at pH 7.5 (Figure 4C). These results demonstrate that Glu180 plays a key role in the pH-mediated switching of substrate specificity of VX-4.

The impact of a single AA substitution at a critical position has been shown in a *Leishmania major* cathepsin B-like protease, in which a Gly residue at the putative S2 pocket provided no detectable proteolytic activity against Z-RR-AMC, while its replacement with Glu restored activity [33]. A similar result was also observed for papain, which exhibited a preference for Phe over Arg at the P2 position, but exhibited cathepsin B-like specificity when the S2 subsite was altered [34]. A cathepsin B-like cysteine protease of *Giardia lamblia* that harbors a Glu residue at the S2 pocket was active against both Z-FR-MCA and Z-LR-MCA [35]. The crystallographic structure of cruzain, an essential cysteine protease of *Trypanosoma cruzi*, demonstrated that the side chain of Glu205 might vary positions and interact with different substrates according to pH and availability of an electrostatically appropriate partner in the S2 pocket [36]. Therefore, the S2 subsite might be intimately involved in the determination of ligand specificity. Although most of the residues delineating the S2 pocket are hydrophobic, a polar residue is present at the pocket's hollow end in some cysteine proteases [37] including VX-4 (Supplementary Figure S1; see also above). The S2 pocket of these enzymes might retain a negative charge at physiologic pH, allowing the capability to bind the polar guanadino group of Arg at the P2 position.

### VX-4 May Exert Its Activity in Maturation of Plasmepsin and Digestion of Erythrocytic Actin, While Having Adjuvant Roles in Hemoglobin Hydrolysis

Comparative analysis revealed that two motifs, the FP2 nose and FP2 arm, specific to the hemoglobin-degrading falcipain homologs, were conserved in VX-4 (Supplementary Figure S1). The FP2 nose interacts with the protease core *via* a highly conserved KEA motif to provide proper folding of the mature protein, while the FP2 arm mediates interaction between the enzyme and hemoglobin [26], [38], [39]. We recognized some differences in the FP2 arm motif of VX-4, in which residues Phe192, Ser194 and Ala198 (numbered from the mature sequence of VX-4) showed different degrees of hydropathy compared to those of other VXs. In addition, Ala198 of VX-4 offered a unique hydrophobic polymorphism, which in structural modeling contributed considerable change in the arm structure (data not shown). These observations suggested that VX-4 may act principally on substrates other than hemoglobin.

**Figure 5:**
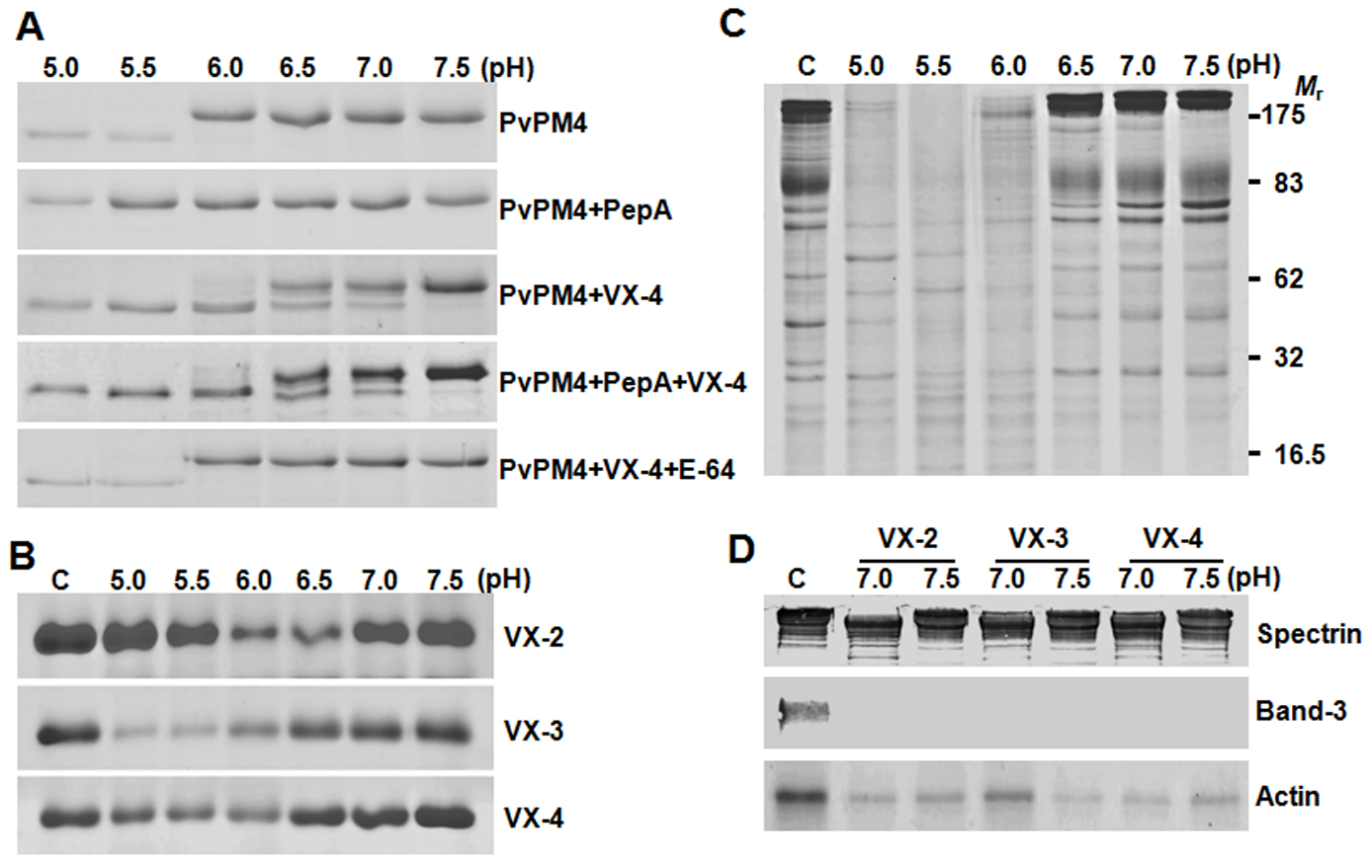
Reactivity of rVX-4 against macromolecular substrates. (**A**) Processing of *P. vivax* plasmepsin 4 (PvPM4) by rVX-4. Recombinant PvPM 4 (20 µg) was incubated with rVX-4 (50 nM) supplemented with 10 mM DTT at different pH values with or without pepstatin A (10 µM) or E-64 (1 µM) for 3 h at 37°C. The reactants were analyzed by 12% SDS-PAGE. (**B**) Comparison of hemoglobinolytic activity of VX-2, VX-3 and VX-4. Native human hemoglobin was incubated with the respective enzymes in appropriate buffers (pH ranges 5.0–7.5) supplemented with 1 mM GSH for 3 h at 37°C, after which resolved by 10% SDS-PAGE. (**C**) Hydrolysis of erythrocyte membrane proteins by rVX-4 at different pHs. Fresh erythrocyte ghosts were incubated with rVX-4 in appropriate buffers (pHs 5.0–7.5) for 3 h at 37°C and reaction products were analyzed by 10% SDS-PAGE. Molecular masses in kDa are shown to the right. (**D**) Western blotting of erythrocyte ghost proteins. The reactions were done at pH 7 and 7.5. The reactants were separated by 10% SDS-PAGE, transferred to a PVDF membrane and probed with specific antibodies against human erythrocyte spectrin (1∶500), band 3 (1∶30000) and actin (1∶1000) followed by horseradish peroxidase conjugated anti-human IgG (1∶1000). The blots were developed with 4C1N. C, control without enzyme.

We assessed whether VX-4 plays a role in PM processing since a recent study has revealed that FPs function as maturases for PMs within the food vacuole of *P. falciparum* [40]. *Plasmodium* species infecting mammals harbored genes for seven PMs (PM4-PM10), of which PM4 orthologs were found in the food vacuole [23]. *P. falciparum* genome encoded additional food vacuole-related proteins, PM1, PM2, and histo-aspartic protease (HAP), although genes orthologous to these proteins genes were not detected in non-falciparum species. [23], [41], [42]. These results suggest that PvPM4 is the major, if not all, plasmepsin targeted into the food vacuole of *P. vivax*. We examined possible roles for VX-4 during maturation of recombinant PvPM4 (rPvPM4), which was expressed in *E. coli*. As shown in Figure 5A, autocatalytic processing of rPvPM4 occurred at acidic pH and, to a less extent, at neutral pH (6.5–7.0). This processing was completely blocked by the aspartic protease inhibitor pepstatin A. This cleavage was significantly accelerated in the presence of VX-4 in a dose- and time-dependent manner (data not shown). In the presence of pepstatin A to block autocatalysis, rVX-4 effectively cleaved rPvPM4 at pH 5.0–7.0, and this process was specifically and significantly inhibited by E-64. These results suggest that VX-4 is a key molecule regulating PvPM4 maturation. Processing may occur during trafficking of the enzymes from endoplasmic reticulum (ER)-derived transport vesicles or the parasitophorous vacuolar space (PVS), where pH is neutral, or in the acidic food vacuole (pH 5.4–5.5) [43], [44]. VX-2/VX-3 might also participate in the processing in the food vacuole.

The major hemoglobinases of *P. falciparum* are targeted into the food vacuole through ER-derived vesicles, but it is unclear whether the ER-derived, protease-containing vesicles fuse with hemoglobin-containing transport vesicles derived from cytosomes, or if they directly contact the food vacuole [43], [45]. The bipartite signals, composed of cytoplasmic, transmembrane and lumenal motifs, were found to be required for trafficking of FP-2 and FP-3 to the food vacuole, and they are conserved in VX-2, VX-3, and VX-4 (Supplementary Figure S1) [39], [46]. The hemoglobinase activity of VX-4 was compared to that of VX-2 and VX-3. pH-dependent and time-lapse analyses demonstrated that the hemoglobinolytic activity of VX-4 was relatively weak. Maximal hemoglobin degrading activity of VX-2, VX-3 and VX-4 was observed between pH 6.0–6.5, 5.0–6.0, and 5.5–6.0, respectively (Figure 5B). Considering their peak activities at different pHs, the action points of different VXs may be temporally segregated during hemoglobin degradation. However, it is unclear whether the biochemical differences between VX-2, VX-3, and VX-4 are most important to foster cooperative action against hemoglobin or to provide activities against different substrates over the course of erythrocytic infection by *P. vivax*.

To consider other potential substrates for VX-4, we examined hydrolytic activity against erythrocyte cytoskeletal proteins (Figures 5C and D). VX-4 cleaved the majority of erythrocytic ghost proteins under acidic conditions (pH 5.0–6.0), whereas some activities were negligible at neutral pH (6.5–7.5). However, VX-2, VX-3, and VX-4 all degraded band-3 (anion exchanger 1, AE1) and actin at neutral pH. The proteolytic activities of VX-4 against erythrocyte actin and band-3 suggest an additional role for the protease in remodeling of erythrocyte cytoskeleton during the process of egress of merozoites from erythrocytes at the conclusion of the parasite erythrocytic cycle. Alternatively, actin degradation may be directly related to hemoglobin transport into the food vacuole, as a recent study showed that actin filament turnover in *P. falciparum* might be essential for both cytostome formation and hemoglobin translocation [44].

### VX-4 Localizes Densely within the Food Vacuoles and Adjacent Areas, as Well as Diffusely in the Cytoplasm in Whole Erythrocytic Stages of *P. vivax*

The major hemoglobinases of *P. falciparum* (FP-2, FP-3 and plasmepsins) are targeted into a food vacuole through the ER-derived vesicles. However, it could not be clearly concluded whether the ER-derived, protease-containing vesicles are fuse with hemoglobin-containing transport vesicles, which were pinched off from cytosomes, or they directly contact with the food vacuole [43], [45]. However, investigations have been highly limited with the *P. vivax* proteins, not only due to low parasitemia in the patients' blood, but also due to failure of experimental maintenance of the parasite. The N-terminal regions of vavapains conserved the characteristic bipartite signal for trafficking to the food vacuole, which included cytoplasmic, transmembrane and lumenal motifs [26] (Supplementary Fig. S1). Alternatively, the proteolytic activities of VX-4 against erythrocytic actin and band-3 might suggest its additional role(s) in the remodeling of erythrocytic cytoskeleton, although the low activity with spectrin, which is one of the major cytoskeletal proteins in erythrocyte, makes it unclear (Figure 5D).

**Figure 6:**
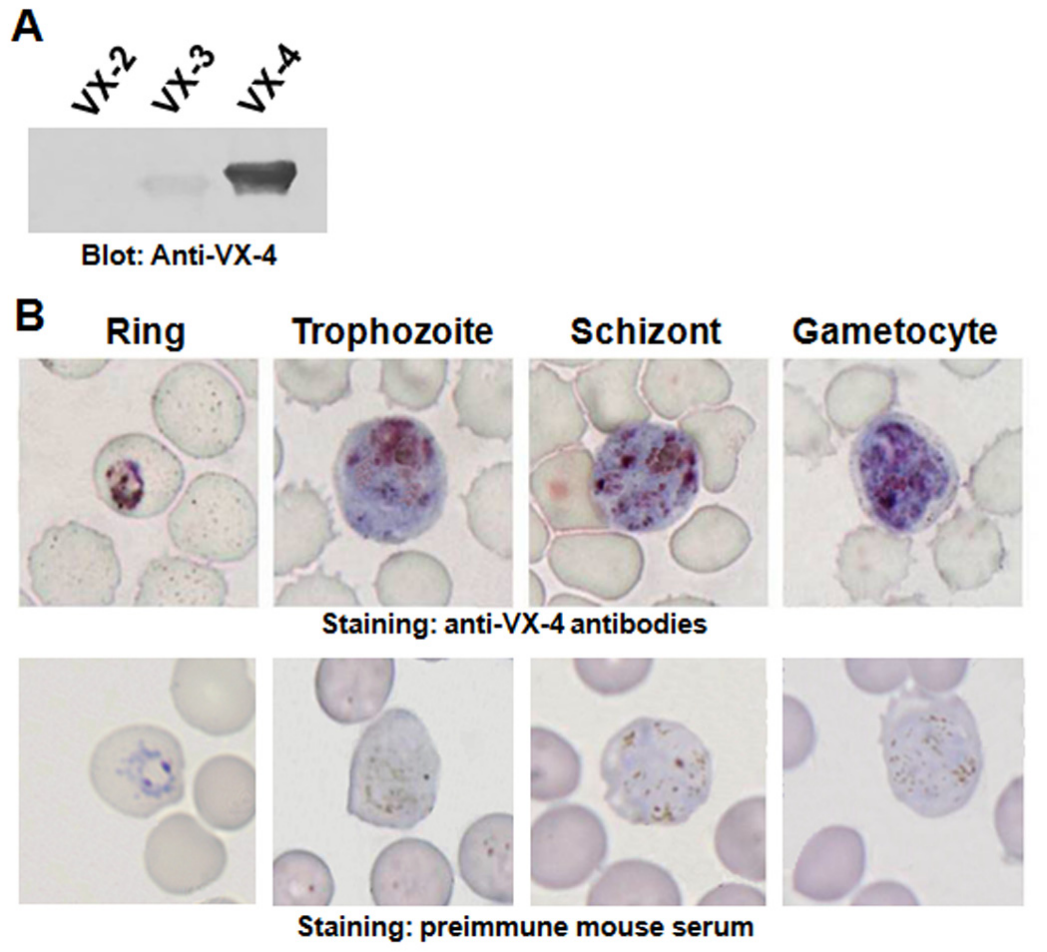
Spatiotemporal Localization of VX-4 by immunocytochemical staining. (**A**) rVX-2, rVX-3 and rVX-4 proteins were separated by 12% SDS-PAGE and transferred to PVDF membrane. The membrane was incubated with anti-rVX-4 (1∶1000 dilutions) for 4 h, with an additional incubation with peroxidase-conjugated anti-mouse IgG (1∶2000 dilutions) for 4 h. The blot was developed using 4C1N chromogen. (**B**) A thin blood smear from a patient with vivax malaria was stained with anti-rVX-4 conjugated with avidin-biotin complex system. The protein was densely labeled in the food vacuoles and adjacent areas, as compared to the staining of the dark hemozoin pigment.

We prepared a mouse antiserum specific to rVX-4, which showed negligible cross-reactions with rVX-2 and rVX-3 as well as erythrocyte proteins (Figure 6A). We assessed the spatiotemporal expression pattern of VX-4. As shown in Figure 6B, VX-4 was shown to be expressed through all of the intraerythrocytic stages of *P. vivax*, from ring to schizont/gametocyte stages. VX-4 localization appeared to be largely limited to the food vacuoles with dark homozoin pigment, while the protein was also labeled diffusely in the parasite cytoplasm. *P. falciparum* FP-3 seemed to have a biological implication(s), which is pivotal to the parasite's survival, in addition to the hemoglobin degradation [46] and showed a distribution pattern similar to that of VX-4 [47]. Given the fact that VX-4 has hydrolytic activity against cytoskeletal proteins, the cytoplasmic distributions of VX-4 and FP-3 might suggest their cytosolic roles such as cytoskeletal remodeling and hemoglobin transportation, which is pivotal for the maintenance of intraerythrocytic stage of the parasites.

### Concluding Remarks

The substrate specificity of proteases depends largely on interactions between a substrate and the enzyme active site. The binding efficiency is greatly affected by the physicochemical micromilieu. The reaction pH may confer substrate preference, as has been seen with cruzain [36]. The pH-dependent substrate switching of VX-4 might be relevant to its multiple biological roles; the protein might function as a maturase of PvPM4 in the plasma membrane or cytosomes at neutral pH, while it participates in the degradation of hemoglobin in the acidic food vacuole. VX-4 might also be involved in cytoskeletal remodeling for the invagination of parasite plasma membrane to form cytostomes and/or the hydrolysis of host proteins to facilitate parasite egress from the erythrocyte. VX-4 thus may be a multifunctional enzyme, performing pivotal functions to ensure parasite survival during the complex life cycle of *P. vivax*. Given the multifunctional activities of VX-4, which are critical for the survival and/or metabolic homeostasis of the parasite, the enzyme might be an attractive target for the development of new antimalarial chemotherapeutics. Work toward further identification of natural substrates and distinct protease functions are currently underway to facilitate a more comprehensive understanding of the biological significance of this enzyme.

## Supporting Information

Figure S1 Multiple alignment of amino acid sequences of vivapain-4 and its homologs in *Plasmodium* genomes. Numbers of amino acids (AAs) in full-length polypeptides are marked at right side of each of the alignments and numerical in parentheses indicates those of mature forms. Dots indicate gaps introduced into the alignment to maximize similarity values. Boxes indicate sequence motifs of interest based on the FP-2 structure. The ERFNIN and GNFD signatures of prodomains are marked by red letters. Shading marks a putative starting position of each mature domain. Red arrow indicates amino acid position corresponding to the N-terminal region of recombinant VX-4. Three AA residues of S2 pocket, which were selected for the mutagenesis experiments, are indicated by dotted red circles. YP-2, yoelipain-2 (XP_726900); BP-2, berghepain-2 (XP_680416); CP-2, chabaupain-2 (AAP43630); VP-2, vinckepain-2 (AAL48319); FP-2, falcipain-2 (XP_001347836); FP-2B, falcipain-2B (XP_001347832); FP-3, falcipain-3 (XP_001347833); KP-4, knowlepain-4 (CAQ39924); VX-4, vivapain-4 (XP_001615272); KP-2, knowlepain-2 (CAQ39926); VX-2, vivapain-2 (XP_001615274); KP-3, knowlepain-3 (CAQ39925); VX-3, vivapain-3 (XP_001615273).
(1.20 MB TIF)

Table S1 Pairwise divergence matrix of plasmodial falcipain homologs (FPs) based on the Jones-Taylor-Thornton model.
(0.03 MB DOC)
