# Supplementary figures and images for: Biochemical Properties of a Novel Cysteine Protease of Plasmodium vivax, Vivapain-4
Source: PLoS Negl Trop Dis. 2010 Oct 12;4(10):e849. doi: 10.1371/journal.pntd.0000849 (PMC2953480; doi:10.1371/journal.pntd.0000849)

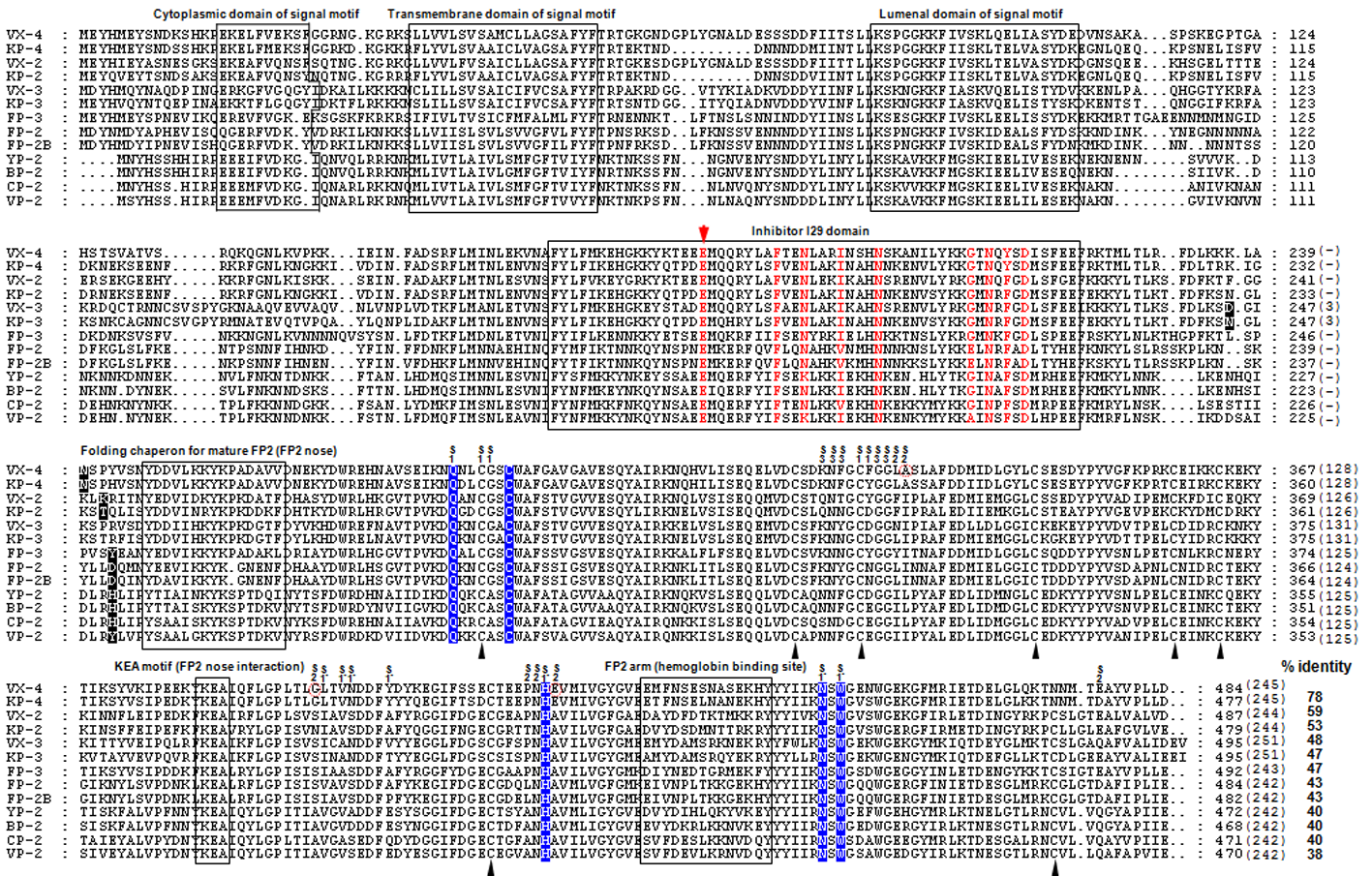

Supplement: Figure S1 — Multiple alignment of amino acid sequences of vivapain-4 and its homologs in Plasmodium genomes. Numbers of amino acids (AAs) in full-length polypeptides are marked at right side of each of the alignments and numerical in parentheses indicates those of mature forms. Dots indicate gaps introduced into the alignment to maximize similarity values. Boxes indicate sequence motifs of interest based on the FP-2 structure. The ERFNIN and GNFD signatures of prodomains are marked by red letters. Shading marks a putative starting position of each mature domain. Red arrow indicates amino acid position corresponding to the N-terminal region of recombinant VX-4. Three AA residues of S2 pocket, which were selected for the mutagenesis experiments, are indicated by dotted red circles. YP-2, yoelipain-2 (XP_726900); BP-2, berghepain-2 (XP_680416); CP-2, chabaupain-2 (AAP43630); VP-2, vinckepain-2 (AAL48319); FP-2, falcipain-2 (XP_001347836); FP-2B, falcipain-2B (XP_001347832); FP-3, falcipain-3 (XP_001347833); KP-4, knowlepain-4 (CAQ39924); VX-4, vivapain-4 (XP_001615272); KP-2, knowlepain-2 (CAQ39926); VX-2, vivapain-2 (XP_001615274); KP-3, knowlepain-3 (CAQ39925); VX-3, vivapain-3 (XP_001615273). (1.20 MB TIF) [file pntd.0000849.s001.tif]
