# Supplementary material for: Biochemical Properties of a Novel Cysteine Protease of Plasmodium vivax, Vivapain-4
Source: PLoS Negl Trop Dis. 2010 Oct 12;4(10):e849. doi: 10.1371/journal.pntd.0000849 (PMC2953480; doi:10.1371/journal.pntd.0000849)
Supplement: Table S1 — Pairwise divergence matrix of plasmodial falcipain homologs (FPs) based on the Jones-Taylor-Thornton model. (0.03 MB DOC) [file pntd.0000849.s002.doc]

**Supplementary Table S1**

**Pairwise divergence matrix of plasmodial falcipain homologs (FPs) based on the Jones-Taylor-Thornton model^a^**

| Group^b^ | 1 | 2 | 3 | 4 |
| --- | --- | --- | --- | --- |
| 1, Rodent FP-1 | **0.377 ± 0.056** | 1.261 ± 0.130 | 3.032 ± 0.480 | 2.735 ± 0.393 |
| 2. Primate FP-1 |  | **0.266 ± 0.035** | 2.840 ± 0.366 | 2.466 ± 0.338 |
| 3. Rodent FP-2 |  |  | **0.271 ± 0.034** | 0.917 ± 0.0112 |
| 4. Primate FP-2^c^ |  |  |  | **0.812 ± 0.078** |

^a^Distance values are presented as mean ± standard error. The standard error was computed by bootstrapping of 1,000 replicates after removing gaps as missing information in a pairwise manner.

^b^The malaria cysteine proteases were categorized into each of the groups based on a phylogenetic analysis (see Fig. 1).

^c^The highly redundant cysteine proteases of *P. falciparum* (FP-2B) and *P. reichenowi* (RP-3) were excluded in the analysis.
